# Rural Re-entry and Opioid Use: Identifying Health-Related Predictors of Relapse Among Formerly Incarcerated Women in Appalachia

**DOI:** 10.13023/jah.0303.03

**Published:** 2021-07-25

**Authors:** Joseph M. Calvert, Megan F. Dickson, Martha Tillson, Erika Pike, Michele Staton

**Keywords:** Appalachia, opioid relapse, community re-entry, rural women, incarcerated women, substance abuse treatment, discharge planning

## Abstract

**Introduction:**

Despite improved knowledge of the health care needs of formerly incarcerated women, there exists a gap regarding the relationship between health, health care access, and relapse among rural women returning to the community during the opioid epidemic.

**Purpose:**

With an emphasis on health care access, this study examined health-related factors associated with opioid relapse among women reentering the community in rural Appalachia.

**Methods:**

As part of a larger study, 400 rural women reporting a history of substance use were recruited from three Appalachian jails in Kentucky. Analyses focused on participants reporting a history of illicit opioid use prior to incarceration, who had also completed follow-up interviews at 6- and 12-months post-release from jail.

**Results:**

Fifty-five percent of participants reported relapse to opioids during the 12-month follow-up period. Compared to those who did not use opioids during this time, women who relapsed reported poorer mental and physical health, as well as encountered more barriers to needed health services. They were also more likely to report a usual source of care. Multivariate regression analyses reveal that, even when controlling for other known correlates of opioid use and relapse to any non-opioid drug during the follow-up period, the number of barriers to health service utilization was a significant predictor of opioid relapse.

**Implications:**

Stakeholders should address the complex reentry needs of women who use opioids in rural Appalachia. This includes examining innovative approaches to reduce extensive barriers to quality health care utilization, such as implementing telehealth for opioid use treatment.

## INTRODUCTION

Women have historically accounted for a minority proportion of the incarcerated population in the United States.^1^ Consequently, the bulk of scholarly attention to incarceration and re-entry experiences, including evidence-informed policy and program implications, has been androcentric.^2^ However, the incarceration rate for women increased at twice that of men during the prison boom (i.e., the period from 1973 to 2007 during which the incarceration rate rose dramatically from a historically stable rate of 161 per 100,000 population to 767 per capita) and remains on the rise despite a recent downward trend in the overall rate.^1^ More recently, a growing movement towards decarceration has yielded commensurately high rates of community re-entry. Moreover, a larger percentage of these reintegrating citizens will be women than at any other timepoint, suggesting the importance of continued resources to support successful re-entry outcomes for women, who have diverse and complex re-entry needs.^2^,^3^

One particular re-entry need among women that has been highlighted in the literature is health services. Existing studies suggest that women re-entering the community following incarceration have unique health needs,^4^ needs that may be intensified among those with a history of drug use.^3^ Opioid use, in particular, is associated with chronic physical or mental illness and disability.^5^ Women, however, often report encountering barriers to needed health services upon release from custody,^6^ despite evidence that access to health services can facilitate more successful re-entry outcomes.^4^ Notable barriers to health services reported by reintegrating citizens include insufficient means to pay for services, lapsed insurance coverage during incarceration, and limited health service options.^7^

Women re-entering to rural communities may be further hindered by wide geographic dispersion of healthcare providers, limited transportation, and financial challenges due to widespread unemployment and economic marginalization.^8^ In Appalachian regions, these individuals also face elevated mortality rates associated with heart disease, cancer, stroke, diabetes, injury, suicide, and opioid overdose.^9^ The prevalence of health outcome disparities in rural Appalachian areas, combined with significant barriers to health services access and engagement, has resulted in an environment with high potential for opioid misuse and diversion.^10^ Widespread chronic pain, lack of economic and educational opportunity, and close-knit, place-based social networks all contribute to risks of substance use and untreated health concerns.^11^ For recently incarcerated rural women, this constellation of issues may be particularly salient as they navigate the complex process of recovery and re-entry,^3^,^12^ which is associated with a heightened risk of drug overdose and death by other means (e.g., cardiovascular disease, homicide, and suicide).^13^

Although untreated health problems are highly correlated with relapse and further criminal justice involvement for individuals re-entering the community,^3^ little is known about the relationship between health, healthcare access, and relapse among rural women returning to the community within the context of the opioid epidemic, particularly in rural Appalachia. Therefore, the current study examines health-related predictors of opioid relapse among rural Appalachian women re-entering the community, while also considering the role of access to health care.

## METHODS

### PARTICIPANTS

As part of a larger study (NIH/NIDA 1R01-DA033866), 400 rural women with a history of drug use were recruited from three rural Appalachian jails in Kentucky. As described in greater depth elsewhere,^14^ participants were randomly selected, screened, and interviewed face-to-face at baseline between December 2012 and August 2015. The final analytic sample for the current study was comprised of participants reporting a lifetime history of illicit opioid use prior to incarceration and who also completed follow-up interviews at both 6- and 12-months post-release from jail (N=342). At follow-up, participants were asked about their mental and physical health, barriers to health services, and service utilization during community re-entry.

### MEASURES

**Demographics**. Sociodemographic measures for this study included age (in years at baseline), education (years of formal education), and employment status (employed at least part-time during the follow-up period).

**Substance use**. Substance use patterns were assessed via a dichotomous indicator of lifetime history of injection drug use, while opioid use severity was measured with the NIDA-Modified Alcohol, Smoking, and Substance Involvement Screening Test (NM-ASSIST). Specifically, participants answered items related to intensity and frequency of use for both street and prescription opioids, as well as a desire to use, life problems attributed to use, and how they and others felt about their use. Risk scores ranged from 0 to 39. An NM-ASSIST score of 4+ for any drug was a criterion for inclusion in the larger study.

**Mental health**. Mental health was measured using the Global Appraisal of Individual Needs (GAIN). Participants reporting symptoms consistent with each mental health problem (depression, anxiety, and post-traumatic stress disorder (PTSD)) were coded dichotomously (yes/no). Utilization of mental health treatment at any time during the 12-month follow-up period was also assessed via dichotomous self-report indicator.

**Physical health**. To assess physical health, participants were asked if they experienced “a lot of physical pain or discomfort” during the follow-up period (yes/no). Additionally, the Miami Health Services Utilization questionnaire was administered to assess health problems and health service utilization. Participants were asked if they had a usual source and if they were currently being treated for a health problem (yes/no). Additional measures included barriers to health service utilization that were dichotomously coded, including: (1) could not afford medical care; (2) no insurance coverage; (3) did not have a way to get there; (4) too far to travel for care; (5) could not get appointment; and (6) right kind of service not available. Finally, the total number of barriers experienced was calculated.

**Outcome variable**. The dependent variable (relapse to opioids) was a dichotomous indicator of self-reported opioid use at any point during the 12-month follow-up study period.

### ANALYTIC PLAN

Analyses conducted in 2019 first separated participants into two groups based on self-reported opioid use during the 12-month follow-up period: those who reported relapse to opioids (n=187) and those who did not (n=155). Then, a series of chi-square tests and t-tests were used to examine differences in demographics, mental and physical health, service utilization, and barriers to health services during community re-entry across the two groups. Finally, multivariate logistic regression models were used to examine health- and service-related predictors of opioid relapse during the follow-up period, while controlling for demographic differences and substance use, including non-opioid relapse. Statistical analyses employed two-tailed tests with an alpha level of 0.05 and were performed using IBM SPSS 24.

## RESULTS

### SAMPLE DESCRIPTION

Overall, participants had an average age of 32.4 years old (SD=7.9), and half (50.6%) had not graduated high school at the time of baseline. The majority were white (98.8%), single (60.2%), and unemployed (72.2%). More than three-quarters (77.2%) reported a history of injection drug use, while the mean opioid NM-ASSIST score was 28.8 (SD=13.2). Fifty-five percent (n=187) of participants self-reported using opioids during the 12-month follow-up period.

### BIVARIATE ANALYSES

Compared to those who did not use opioids during the follow-up period (n=155), participants who relapsed were significantly younger (t(300.03)=2.43, p=0.016), less educated (t(340)=2.62, p=0.009), and less likely to be employed (x2(1, N=342)=21.11, p<0.001). Those who relapsed to opioid use during the follow-up period also reported poorer mental and physical health than those who did not report any opioid use (Table 1). Specifically, they were significantly more likely to endorse criteria for depression (x2(1, N=342)=11.34, p=0.001), anxiety (x2(1, N=342)=19.07, p<0.001), and PTSD (x2(1, N=342)=10.70, p=0.001). Those who used opioids during the follow-up period also were more likely to report experiencing physical pain and discomfort (x2(1, N=342)=11.62, p=0.001) during the follow-up period. Individuals who relapsed during the follow-up period reported a higher mean opioid NM-ASSIST score (t(307.20)=−3.94, p<0.001) and were more likely to report a history of injection drug use (x2(1, N=342)=23.31, p<0.001).

Access to care also differed between groups. Individuals who relapsed to opioid use during the follow-up period encountered significantly more barriers to needed health services (t(340)=−2.80, p=0.005) compared to those who did not return to opioid use, including not having transportation (x2(1, N=342)=6.04, p=0.014), long travel distances (x2(1, N=342)=6.37, p=0.012), and being unable to get an appointment (x2(1, N=342)=8.57, p=0.003). However, those who returned to opioid use were more likely to report a usual source of care when sick or for health consultation (x2(1, N=342)=5.19, p=0.023).

### MULTIPLE REGRESSION ANALYSES

Two logistic regression models were used to identify predictors of opioid relapse (Table 2). The first model focused on health-related predictors, revealing that experiencing pain more than doubled the odds of returning to opioid use during the follow-up (p=0.009) when controlling for demographic differences and substance use. Having a history of injection drug use (p<0.001), opioid use severity (NM-ASSIST; p=0.040), and relapsing to any other non-opioid drug (p<0.001) also increased the likelihood of opioid relapse. On the other hand, being older (p=0.008), more educated (p=0.017), and employed (p=0.005) decreased the likelihood of opioid relapse. The second logistic regression model incorporated service barriers and having a source of care. Results showed that experiencing pain was no longer a significant predictor of opioid relapse when including access to care in the model and controlling for demographic differences and substance use. For each additional barrier to care reported, the likelihood of relapsing to opioid use increased by 22% (p=0.043), though having a usual source of care also increased the odds of relapsing (AOR=1.77; p=0.046).

## IMPLICATIONS

With a focus on healthcare access, this study investigated factors associated with opioid relapse during community re-entry in rural Appalachia among a sample of women. Results indicate that women who relapsed to opioid use self-reported having more mental and physical health problems, as well as having a usual source of care. However, these participants experienced greater difficulty in accessing needed health services. Overall, these findings suggest that women who relapsed to opioids may be self-medicating to manage pain, but access to care may impact self-medication. This may signal important implications, including examining approaches to improving access to quality health care in rural Appalachia, particularly for opioid-using women re-entering the community after jail.

Barriers to care were often related to travel and resource availability, like physical distance, inadequate transportation, and limited appointment availability. This finding reaffirms prior research on substance use and healthcare utilization, which identifies scarce access as a principal factor associated with low utilization rates.^15^ Overall, the supply of specialty physicians in Appalachia is about 28% lower than the national average.^9^ Central Appalachia (inclusive of Eastern Kentucky) stands out among an already disadvantaged region in this metric, having only 54 specialists per 100,000 population (i.e., fully 65% lower than the national average). Further, Central Appalachia also has approximately 33% fewer primary care physicians per capita than the rest of the nation.^9^

Telehealth is a promising service modality that may alleviate availability and access barriers for this population of women who use substances.^16^ Broadly, telehealth may refer to “(1) telemedicine or synchronous videoconferencing between clinicians and patients in separate locations; and (2) mobile health, involving telephone, text, or web-based interventions.”^16^ This service modality allows patients to access a large pool of physicians and clinicians across potentially great geographic distances, easing access issues for women re-entering the community. While Central Appalachia is characterized as having a dearth of primary care physicians, it is encouraging that, with 137 mental health providers per capita, the subregion has a larger proportion than Appalachia at large.^9^ In this case, telehealth could be used as a tool by the existing primary care and mental healthcare infrastructure in Appalachia to enhance access to specialized treatment for reintegrating women.^9^,^15^ This is an important consideration for future scientific inquiry because implementing telehealth for those who use substances in rural areas is not without its challenges, such as legislative and regulatory impediments (e.g., reimbursement and prescription restrictions), technological and infrastructure-related limitations, and clinician reticence.^16^

Scholars suggest initiating telehealth in correctional facilities prior to release, particularly for those with a history of substance use.^17^ Though the implementation of telehealth in correctional settings currently varies widely, it is generally associated with patient satisfaction, positive clinical outcomes, and reduced costs over time.^17^ However, there are also a variety of perceived barriers associated with telehealth delivered in this context, such as initial cost of equipment and maintenance, as well as resistance from staff.^18^ Future research should explore the utility of initiating telehealth while incarcerated and continuing it upon release for this population.

It is also important to note that barriers to care experienced by those re-entering the community from jail may be exacerbated by the current COVID-19 crisis. Studies have linked pandemic-related social restrictions to a variety of negative outcomes, such as increased financial strain, elevated rates of perceived stress and depression symptoms, and decreased healthcare utilization among Appalachian samples.^19^ With their unique re-entry needs, women may be especially negatively impacted in this regard. For example, Staton and colleagues report elevated perceptions of social isolation among their sample of Appalachian women during the pandemic.^20^ Considering this, coupled with mounting pressure for large-scale decarceration in response to the COVID-19 pandemic,^21^ it is particularly timely and essential that health and criminal justice resources focus on supporting women as they are released from jail into rural areas. Future research should specifically examine the ways in which re-entry processes and experiences may evolve for women in rural areas in a post-COVID social environment.

Another noteworthy finding is that relapse to opioids was positively associated with having a usual source of care. While unanticipated and perhaps confounding upon initial reflection, this may indicate that a lack of accessible healthcare providers is only one piece of a complex puzzle. One potential explanation for this finding is that these women may have chronic and/or severe health issues, for which they self-medicate to supplement their usual source of care. For example, while some women re-entering the community from jail have a usual source of care, they may experience barriers to adequately utilizing this resource, thus they rely on self-medication. Another explanation may be that stigma and suspicion among rural healthcare providers regarding people who use substances, particularly those who use opioids, affects the quality of physician–patient interaction and the level of care provided, including a reluctance to prescribe medication that could potentially be diverted (e.g., buprenorphine).^22^ Despite a paradigm shift towards treating substance use disorder and addiction as a disease, those who use substances continue to face social stigma in the form of stereotyping (e.g., perceptions of dangerousness, immorality, unpredictability, and culpability); general negative emotional reactions (e.g., fear and anger); and discrimination (e.g., social isolation and decreased intention to help).^23^ For the current sample, recent involvement with the criminal justice system may intensify this stigma, creating an additional barrier to care worthy of scholarly attention. Though study analyses do not necessarily indicate a causal relationship between having a usual source of care and relapse to opioids, future research should consider a more in-depth examination of this association, including the possible influence of mediating variables.

These explanations further highlight the importance of reducing barriers to accessing care, as well as point towards the potential of telehealth as a bridge to care for women who use opioids. This is especially salient given the following considerations: (1) drug-related overdose mortality rates have increased steadily over the past couple decades, with opioids and synthetic opioids accounting for a growing share in recent years (e.g., more than two-thirds of overdose deaths in 2017)^24^; (2) the first 2 to 4 weeks immediately following release from custody present a critical period of heightened risk of death for former inmates, with overdose being the leading cause of death (i.e., 129 times more likely than the general population)^13^; and (3) injection drug/opioid use is associated with the spread of bloodborne illnesses, like hepatitis C.^25^

The present findings should be interpreted in light of several limitations. This study relied on a self-reported indicator of opioid relapse, which is a potentially sensitive and illegal behavior. Additionally, while interviews were completed in a private setting within the jails or community, some participants may have been concerned about the confidentiality of their responses. Therefore, the risk of social desirability influencing responses cannot be ruled out, which poses a threat to validity and reliability. As such, caution should be exercised when considering causality and prediction in the observed relationships between study variables. Participants were recruited from three rural Appalachian jails, which may limit the generalizability of results to those reintegrating from prisons or those re-entering into rural, non-Appalachian communities. Finally, current analyses did not incorporate a measure of relationship trauma or partner violence for the women in this sample, despite existing evidence of an association between trauma and substance use.^3^ Future studies should consider this potential connection as it could also impact women’s healthcare needs.

Despite these limitations, the current study enhances scholarly understanding of the factors that impact opioid relapse among formerly incarcerated women re-entering the community from jail in rural Appalachia. In response to the results of this study, policymakers and healthcare professionals should: (1) focus discharge/re-entry resources to support the unique needs of women re-entering rural communities, particularly as the social landscape changes while the COVID-19 pandemic continues to evolve (e.g., SARS-CoV-2 Delta); and (2) examine innovative ways to improve health status and erode barriers to healthcare utilization among this population. This includes exploring the feasibility of permanently lifting applicable legislative, regulatory, and reimbursement restrictions, as well as addressing other social and technical challenges (e.g., telecommunications infrastructure), that impede the implementation of telehealth services in the Appalachian region.

**SUMMARY BOX**

**What is already known on this topic?** Existing studies suggest that justice-involved women with a history of drug use have greater healthcare needs, and despite evidence indicating that having access to health services during re-entry can facilitate more successful outcomes, many report encountering barriers to needed health services upon release from custody.

**What is added by this report?** There is a limited understanding of the relationship between health, healthcare access, and relapse among justice-involved rural Appalachian women returning to the community after a period of incarceration, particularly within the context of the opioid epidemic. This report provides evidence of the amplified mental and physical healthcare needs of women who relapse to opioid use during community re-entry and the difficulty they experience in accessing needed health services.

**What are the implications for future research?** Results highlight the importance of supporting the health needs of rural Appalachian women with a history of opioid use who are re-entering the community following incarceration, as well as addressing the far-reaching barriers to healthcare utilization they face. Further research is needed on improving access to quality health care for women who use opioids, which may include utilizing telehealth as a bridge to care and examining the role of stigma as a barrier to care.

## Figures and Tables

**Table 1:** Health, Healthcare Utilization, and Barriers to Care by Opioid Relapse During 12-month Follow-up (N=342)

Variable	Relapse to Opioids (n=187)	Did not Relapse to Opioids (n=155)
**Mental Health – GAIN**		
% Major Depressive Disorder***	58.3%	40.0%
% Generalized Anxiety Disorder***	33.2%	12.9%
% Post-traumatic Stress Disorder***	61.0%	43.2%
% received MH treatment in the past 12 months	28.3%	29.0%
**Physical Health**		
% had a lot of physical pain/discomfort***	57.2%	38.7%
% currently being treated for a health problem	40.1%	41.9%
% had a usual source of care*	62.6%	50.3%
**Substance Use**		
Mean opioid NM-ASSIST score (SD)***	31.3 (12.1)	25.7 (13.9)
% ever injected any drug in lifetime***	87.2%	65.2%
**Barriers to Care**		
Could not afford medical care	29.4%	24.5%
No insurance coverage	29.9%	25.8%
No way to get there/transportation*	39.0%	26.5%
Too far to travel for care*	20.3%	10.3%
Could not get an appointment**	13.9%	4.5%
Right kind of service not available	10.7%	6.5%
Total # of barriers experienced**	1.4	1.0

* *p* ≤ 0.05,

** *p* ≤ 0.01,

*** *p* ≤ 0.001;

*GAIN* Global Appraisal of Individual Needs, *NM-ASSIST* NIDA-Modified Alcohol, Smoking, and Substance Involvement Screening Test

**Table 2:** Correlates of Opioid Relapse during 12-month Follow-up (N=342)

	Model #1	Model #2
Variable	Adj. Odds Ratio (CI 95)	Adj. Odds Ratio (CI 95)
Age	0.95** **(0.92–0.99)**	0.95* **(0.92–0.99)**
Education (years)	0.86* **(0.76–0.97)**	0.86* **(0.76–0.97)**
Employed at least part time	0.42** **(0.23–0.76)**	0.46* **(0.25–0.86)**
Lifetime history of injection	3.30*** **(1.70–6.43)**	3.60*** **(1.82–7.14)**
Relapsed to any non-opioid drug	5.57*** **(3.25–9.54)**	6.22*** **(3.56–10.87)**
Street/RX opioid score (NM-ASSIST)	1.02* **(1.01–1.04)**	1.02* **(1.00–1.04)**
Had a lot of physical pain	2.13** **(1.21–3.76)**	1.76 (0.98–3.14)
Any MH Problem (GAIN)	1.13 (0.64–1.99)	0.90 (0.50–1.63)
# of barriers to health care	—	1.22* **(1.01–1.49)**
Usual source of health care	—	1.77* **(1.01–3.11)**

*Note*: Significant associations are bolded.

* *p* ≤ 0.05,

** *p* ≤ 0.01,

*** *p* ≤ 0.001;

*NM-ASSIST* NIDA-Modified Alcohol, Smoking, and Substance Involvement Screening Test, *GAIN* Global Appraisal of Individual Needs
